# Glutamine Regulates Gene Expression Profiles to Increase the Proliferation of Porcine Intestinal Epithelial Cells and the Expansion of Intestinal Stem Cells

**DOI:** 10.3390/ani13182917

**Published:** 2023-09-14

**Authors:** Min Zhu, Weiming Lai, Lewen Yao, E Xu, Xiang Chen, Yi-yu Zhang, Xiang-Guang Li

**Affiliations:** 1 Laboratory of Animal Genetics, Breeding and Reproduction in the Plateau Mountainous Region, Ministry of Education, College of Animal Science, Guizhou University, Guiyang 550025, China; mzhudky@gzu.edu.cn (M.Z.); exu@gzu.edu.cn (E.X.); xchen2@gzu.edu.cn (X.C.); 2 Institute of Animal Nutrition and Feed Science, Guizhou University, Guiyang 550025, China; 3 Department of Pharmaceutical Engineering, School of Biomedical and Pharmaceutical Sciences, Guangdong University of Technology, Guangzhou 510006, China; 2112112001@mail2.gdut.edu.cn (W.L.); yvleshu@163.com (L.Y.)

**Keywords:** intestinal stem cells, intestinal epithelium, glutamine, gene expression profile, minichromosome maintenance protein, transcriptomics

## Abstract

**Simple Summary:**

The intestinal epithelium is one of the tissues that renews the fastest in the body. This process is driven by intestinal stem cells and is greatly influenced by adenosine triphosphate supply. Glutamine is the preferred energy substrate for the intestinal epithelium, unlike most tissues in the body that prefer glucose. However, there needs to be more understanding of how glutamine affects gene expression in the intestinal epithelium. To address this gap, this study aimed to identify the essential genes and signals involved in glutamine-induced growth of intestinal epithelial cells by exploring gene expression profiles. The results showed that glutamine might upregulate deoxyribonucleic acid replication licensing factors, increasing proliferation-related signaling and suppressing inflammation-related pathways. Consequently, this mechanism leads to the proliferation of intestinal epithelial cells and the expansion of intestinal stem cells. Understanding these crucial targets provides new insight into regulating the homeostasis of the intestinal epithelium, particularly in porcine production.

**Abstract:**

The intestinal epithelium is known for its rapid self-renewal, and glutamine is crucial in providing carbon and nitrogen for biosynthesis. However, understanding how glutamine affects gene expression in the intestinal epithelium is limited, and identifying the essential genes and signals involved in regulating intestinal epithelial cell growth is particularly challenging. In this study, glutamine supplementation exhibited a robust acceleration of intestinal epithelial cell proliferation and stem cell expansion. RNA sequencing indicated diverse transcriptome changes between the control and glutamine supplementation groups, identifying 925 up-regulated and 1152 down-regulated genes. The up-regulated DEGs were enriched in the KEGG pathway of cell cycle and GO terms of DNA replication initiation, regulation of phosphatidylinositol 3-kinase activity, DNA replication, minichromosome maintenance protein (*MCM*) complex, and ATP binding, whereas the down-regulated DEGs were enriched in the KEGG pathway of p53 signaling pathway, TNF signaling pathway, and JAK-STAT signaling pathway and GO terms of inflammatory response and intrinsic apoptotic signaling pathway in response to endoplasmic reticulum stress. Furthermore, GSEA analysis revealed a significant up-regulation of the cell cycle, DNA replication initiation, ATP-dependent RNA helicase activity, and down-regulation of the TNF signaling pathway. The protein–protein association network of the intersecting genes highlighted the significance of DNA replication licensing factors (*MCM3*, *MCM6*, and *MCM10*) in promoting intestinal epithelial growth in response to glutamine. Based on these findings, we propose that glutamine may upregulate DNA replication licensing factors, leading to increased PI3K/Akt signaling and the suppression of TNF, JAK-STAT, and p53 pathways. Consequently, this mechanism results in the proliferation of porcine intestinal epithelial cells and the expansion of intestinal stem cells.

## 1. Introduction

The intestinal epithelium plays a critical role in the digestion, absorption, and metabolism of nutrients and drugs, as well as protection against external pathogens. It consists of a single layer of various epithelial cells, such as absorptive enterocytes, mucus-producing goblet cells, enteroendocrine cells that release gastrointestinal hormones, Paneth cells secreting antimicrobial peptides, and M cells responsible for antigen sampling [1,2]. The intestinal epithelium, powered by intestinal stem cells (ISCs) located at the base of crypts, is one of the most dynamic tissues in the body, exhibiting a remarkable cellular turnover rate of 3–5 days [3]. This renewal process is sustained by the self-renewal, differentiation, maturation, and migration of ISCs and derived cells. ISC-derived progenitors migrate from the crypt base to the villi tips, undergoing differentiation and maturation into functional cell types. Additionally, when cells are impaired or aged, they are swiftly shed, and neighboring cells rapidly seal the gaps to maintain the integrity of the intestinal epithelium [3,4]. Thus, the homeostasis and integrity of the intestinal epithelium are critical for gut health.

Glutamine, a versatile amino acid found abundantly in proteins, serves as a nutrient and plays multiple essential roles in the host. It is also the most abundant free alpha-amino acid in humans, accounting for 20–25% of circulation-free amino acids with a concentration of 0.5–0.8 mM in arterial plasma [5]. In mammalian cells, glutamine is vital in supplying carbon and nitrogen to fuel biosynthesis, which is essential for the growth and healing of intestinal cells [6]. Most of the glutamine and almost all glutamate in the lumen are converted into α-ketoglutarate to generate ATP by intestinal cells [7,8]. Current knowledge emphasizes that glutamine enhances intestinal stem cell-mediated small intestinal epithelial development via Wnt signaling [9]. Moreover, environmental glutamine restriction can also augment Wnt signaling to promote stemness and lead to adenocarcinoma formation via decreasing intracellular alpha-ketoglutarate levels [10]. Hence, maintaining a proper supply and metabolism of glutamine and glutamate is vital for the renewal and functional homeostasis of the intestinal epithelium. However, the current limited knowledge regarding the influence of glutamine on gene expression profiles in the intestinal epithelium significantly hampers the potential application of glutamine-based nutritional interventions for promoting intestinal health.

Gastrointestinal health issues and related problems are major economic costs in the pig production industry. Our previous studies confirm several signals, including excitatory amino acid transporter 3, the mTOR/S6K1 pathway, the IR/IRS/PI3K/Akt pathway, and the Frizzled7/β-catenin pathway, are involved in the glutamate-induced proliferation of porcine intestinal epithelial cells and the expansion of intestinal stem cells [11,12,13,14]. In most cells, glutamine is rapidly metabolized into glutamate by the enzyme glutaminase. Glutamine is the most abundant extracellular amino acid, while glutamate is the most abundant intracellular amino acid in humans [5]. Therefore, given the results of previous studies on glutamate, this study aimed to determine the effects of glutamine on the growth and gene expression profiles of porcine intestinal epithelium. Furthermore, hub genes and pathways were also explored using bioinformatics.

## 2. Materials and Methods

### 2.1. Culture and Treatment of IPEC-J2 Cells

IPEC-J2 cells were maintained in a growth medium (DMEM with 10% FBS, 100 U/mL penicillin, and 100 μg/mL streptomycin) at 37 °C in a 5% CO_2_ incubator (Shellab, Cornelius, OR, USA). The cells were cultivated in the growth medium for 12 h before being exposed to EBSS (H2020, Solarbio, Beijing, China) and a vitamin mixture (11120052, Thermo Fisher, MA, USA) for 2 h. Glutamine (#G8540, Sigma-Aldrich, St. Louis, MO, USA) was dissolved in a glutamine-free medium (SH30285.01, Cytiva HyClone, Logan, UT, USA). For glutamine supplementation experiments, cells in a 96-well plate were exposed to a series of glutamine concentrations (0 mM, 0.5 mM, 1 mM, 2 mM, 4 mM, and 5 mM) for 6 h to determine the optimal culture concentrations. To evaluate the proliferation of IPEC-J2 cells, the MTT assay was used, as described previously [13]. Briefly, each well was added with 20 μL of 3-(4, 5-dimethylthiazol-2-yl)-2, 5-diphenyltetrazolium bromide (MTT) solution (Sigma, St. Louis, MO, USA), followed by careful removal of the supernatant after 4 h. Subsequently, 150 μL of dimethyl sulfoxide (DMSO) was added to each well. Finally, the optical density (OD) values (8 wells/group) were determined using a microplate reader at a wavelength of 490 nm.

To evaluate the proliferation of IPEC-J2 cells, the MTT assay was used, as described in [13]. Each well was treated with 20 μL of MTT solution (Sigma, St. Louis, MO, USA), followed by careful removal of the supernatant after 4 h. Finally, 150 μL of dimethyl sulfoxide (DMSO) was added to each well.

### 2.2. Culture and Treatment of Intestinal Organoids

Intestinal crypts were isolated and cultured as described previously [15]. A 10 cm section of the jejunum from a 7-day-old piglet (Duroc × Landrace × Large White) was isolated and removed from the villi using a glass slide after flushing with ice-cold phosphate-buffered saline (PBS). We then cut the intestinal segments into pieces that were 3–5 mm long, washed them three times with ice-cold soaking buffer (PBS containing 5 mmol/L EDTA, 1.5 mmol/L DTT, and 10 μmol/L Y27632), and placed them in a 50 mL conical tube filled with 25 mL of soaking buffer. The tube was rotated at 200 rpm for 30 min in an ice bath, repeated three times. Next, we manually shook the tube for a period of 10 min, with an average of 2–3 shakes per second. After washing three more times with incubation buffer, the tube was shaken manually for 5 min at a rate of 5–10 shakes per second. We transferred the medium to a new tube and allowed it to precipitate in an ice bath for 5 min. Then we collected the crypts at the bottom of the tube for further study.

Crypts from the jejunum of 7-day-old piglets (Duroc × Landrace × Large White) were embedded in Matrigel at a concentration of 2 crypts/μL Matrigel and seeded into 48-well cell culture plates at 25 μL/well. This allowed for approximately 50 crypts to be seeded in each well. A porcine intestinal stem cell culture medium without GlutaMax (WRN-conditioned medium supplemented with 100 U/mL penicillin, 100 μg/mL streptomycin, 1 × N2, 1 × B27, 10 mmol/L HEPES, 1 mmol/L N-acetyl-cysteine, 10 μmol/L Y27632, and 10% FBS) was added to the control group, while the glutamine supplementation group was supplemented with 2 mM glutamine, as determined by the results with IPEC-J2 cells in this study and an equivalent concentration of glutamate in our previous study [12]. The expansion of intestinal organoids was determined by organoid forming efficiency (5 wells per group), organoid budding efficiency (5 wells per group), and EdU incorporation assay (5 representative organoids per group were calculated).

The organoid-forming efficiency was calculated as the ratio of the colony number to the number of crypts seeded. The organoid budding efficiency was calculated as the percentage of organoids with 2 or more buds over the colony number. In addition, an EdU assay was performed using the Cell-Light EdU Apollo 567 In Vitro Imaging Kit (RiboBio, Guangzhou, China) following the provided instructions. Images were captured using an inverted fluorescence microscope (Ti2-U, Nikon, Tokyo, Japan) and a confocal microscope (Ti2, Nikon, Tokyo, Japan). To measure the fluorescence signal intensity (5 organoids per group), we used the ImageJ software (version 1.8.0 112, National Institutes of Health, Bethesda, MD, USA) and set the mean value in the control group as one. The examiner was not aware of the treatment status of the organoids being examined.

### 2.3. RNA Extraction and cDNA Library Construction

Total RNA was extracted using a Trizol reagent kit (Invitrogen, Carlsbad, CA, USA) following the manufacturer’s protocol. RNA quality was assessed on an Agilent 2100 Bioanalyzer (Agilent Technologies, Palo Alto, CA, USA) and confirmed by RNase-free agarose gel electrophoresis. Subsequently, eukaryotic mRNA was enriched using Oligo(dT) beads. The enriched mRNA was then fragmented into short fragments using a fragmentation buffer and reverse transcribed into cDNA using the NEBNext Ultra RNA Library Prep Kit for Illumina (NEB #7530, New England Biolabs, Ipswich, MA, USA). The resulting double-stranded cDNA fragments were end repaired, an A base was added, and they were ligated to Illumina sequencing adapters. The ligation reaction was purified with AMPure XP Beads (1.0×). Ligated fragments underwent size selection through agarose gel electrophoresis and were amplified by polymerase chain reaction (PCR). Finally, the resulting cDNA library was sequenced using the Illumina Novaseq6000 platform.

### 2.4. Transcriptomics Analysis

To detect the intrinsic repeatability and outliers of the samples, we clustered and visualized the two types of IPEC samples using principal component analysis (PCA). This analysis was performed using the prcomp function in the Vienna (V 4.1.1) R package.

To identify significant differentially expressed genes (DEGs) between the control group and the glutamine treatment group, we utilized the “limma” (V3.54.2) R package. We used the false discovery rate (FDR) to adjust the *p* value, defined as the q value. The DEGs were filtered out based on a q value < 0.05. For up-regulated expression, we defined it as log2FC ≥ 1 with a *p* value < 0.05; for down-regulated expression, we described it as log2FC ≤ −1 with a *p* value < 0.05. Furthermore, the top 30 DEGs were selected based on their ranked fold change |log2FC|.

Next, we conducted a functional enrichment analysis on the DEGs. Initially, the DEGs were annotated using the ‘org.Hs.eg.db’ (V3.14.0) R package to convert gene symbols to ENTREZID. Subsequently, we performed gene ontology (GO), Kyoto encyclopedia of genes and genomes (KEGG) analysis, and gene set enrichment analysis (GSEA) using the ‘clusterProfiler’ (V4.2.2) R package. Finally, the results were visualized using bubble charts.

### 2.5. Protein–Protein Interaction (PPI) Analysis

The genes at the intersection of six crucial pathways were analyzed in PPI. Protein interaction information with a combined score > 0.7 was visualized using Cytoscape (V3.9.1) and its plugin CytoNCA (V2.1.6).

### 2.6. Statistical Analysis

The data are presented as the mean ± SEM. The data were analyzed using SPSS software version 20.0 (SPSS Inc., Chicago, IL, USA). Statistical analysis was conducted using one-way ANOVA for IPEC-J2 cell proliferation and Student’s *t*-test for other experiments. Significance was set at *p* < 0.05.

## 3. Results

### 3.1. Glutamine Accelerates Porcine Intestinal Epithelial Cell Proliferation and Stem Cell Expansion

**Figure 1:**
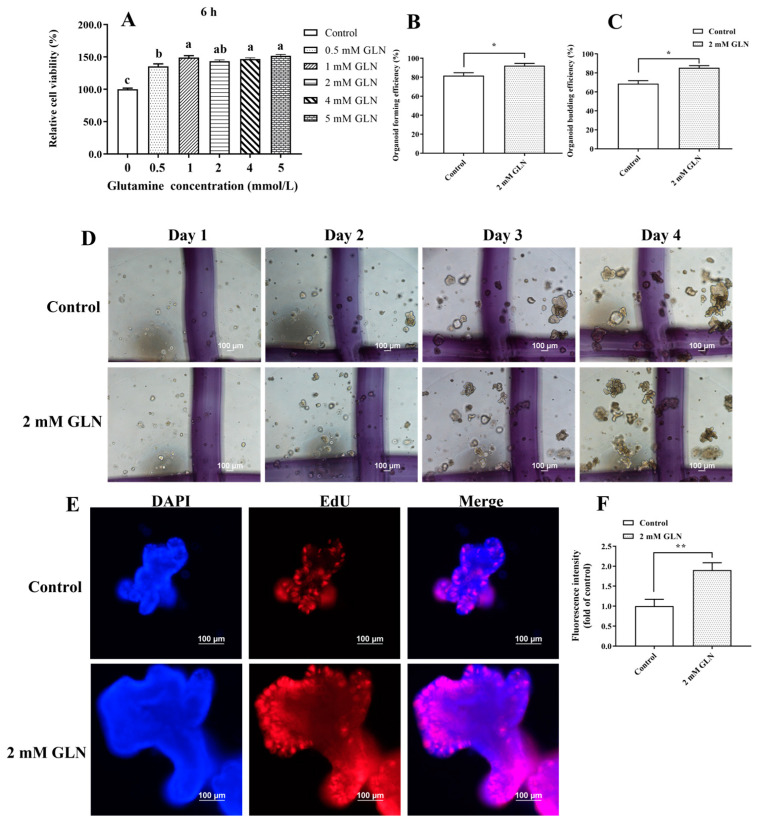
Ex vivo glutamine administration increases the activity of intestinal organoids. (**A**) MTT assay was used to detect the effect of different concentrations of glutamine on IPEC proliferation. The results are expressed as mean ± SEM (*n* = 8 wells per group). Statistical significance was determined by one-way ANOVA. Different lowercase letters indicate significant differences (*p* < 0.05). (**B**,**C**) Organoid-forming efficiency and budding efficiency were measured on day 4. The results are expressed as mean ± SEM (*n* = 5 wells per group, * *p* < 0.05). Statistical significance was determined by Student’s *t*-test. (**D**) Representative images of intestinal organoids cultured from the additive-free culture medium group and the 2 mM glutamine treatment group on days 1, 2, 3, and 4 (40× magnification). (**E**,**F**) Ex vivo administration of glutamine enhanced the mitosis of intestinal stem cells, as indicated by the increasing fluorescence intensity (FI) of EdU (calculated as FIs normalized to the mean FI of the control group). Representative images of intestinal organoids stained with the EdU reagent are shown in the control and 2 mM glutamine groups. The results are expressed as mean ± SEM (*n* = 5 organoids per group, ** *p* < 0.01). Statistical significance was determined by the Student’s *t*-test.

The effects of glutamine supplementation on the proliferation of IPEC-J2 cells and the expansion of porcine intestinal stem cells were explored. The MTT results indicated that 0.5 to 5 mmol/L glutamine supplementation significantly increased (*p* < 0.05) IPEC-J2 proliferation (Figure 1A). Subsequently, crypts in the jejunum of piglets were isolated and cultivated in Matrigel to form three-dimensional intestinal organoids. A concentration of 2 mmol/L glutamine was applied to evaluate its effects on the expansion of porcine intestinal stem cells. Interestingly, glutamine supplementation exhibited a robust promotion of intestinal organoid expansion (Figure 1D), as presented by increased (*p* < 0.05) organoid forming efficiency and organoid budding efficiency at day 4 after the glutamine treatment (Figure 1B,C). To further assess the proliferative ability of intestinal stem cells, intestinal organoids were collected and analyzed with an EdU incorporation assay. Our results showed that glutamine supplementation exhibited significantly higher levels of EdU-labeled mitotic activity than the control group (Figure 1E,F), indicating that glutamine accelerates the proliferation of intestinal stem cells by enhancing mitosis. Thus, these results prove that the administration of glutamine exhibited a robust acceleration of intestinal epithelial cell proliferation and stem cell expansion.

### 3.2. Glutamine Regulates Gene Expression Profiles in the Porcine Intestinal Epithelial Cells

**Figure 2:**
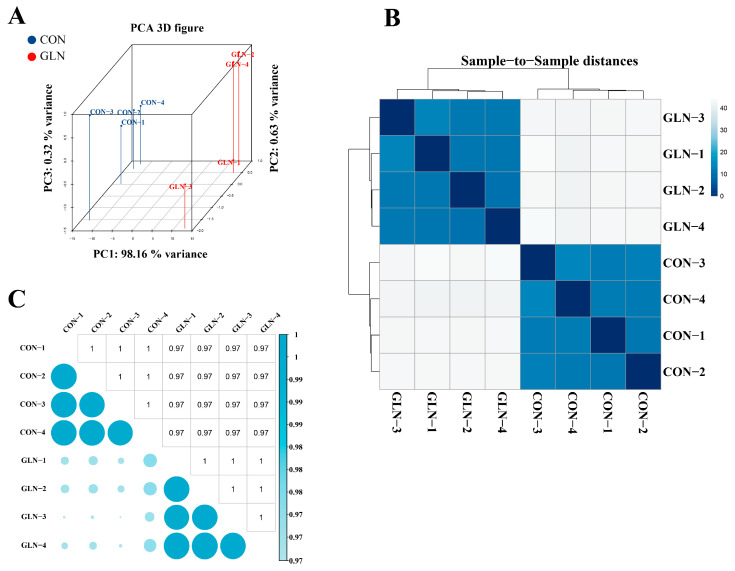
Gene expression profiles between the control and 2 mM glutamine groups. (**A**) Principal component analysis of the groups based on log2(FMPK + 1) corrected expression data. (**B**) Distances within each sample. (**C**) Unbiased hierarchical clustering heat map based on Pearson’s correlation coefficient for 16,201 identified genes. The ordinate and coordinates represent sample names. Color intensity indicates the correlation between samples, with dark blue indicating a high correlation.

To examine the transcriptome changes and biological clustering across the control and glutamine supplementation groups, principal component analysis (PCA) was conducted on gene expression in the eight samples. The PCA analysis revealed that the first three main components explained 99.11% of the variance, with 98.16%, 0.63%, and 0.32% of the PC1, PC2, and PC3 variances, respectively (Figure 2A). Furthermore, we measured the distances between the control and glutamine supplementation groups, indicating significant heterogeneity (Figure 2B). Notably, the groups were distinctly clustered into separate entities, suggesting differing transcriptome profiles.

To further investigate the transcriptome profiles within each group, we performed a hierarchical clustering analysis based on inter-sample correlations. The heatmap visualized the sample correlations, with a deeper blue indicating a higher correlation. The eight samples were represented as columns and rows, classified by their respective groups. Our findings demonstrated a clear distinction between the control and glutamine supplementation samples, with a low correlation observed (Figure 2C). These results indicate diverse transcriptome changes between the groups.

### 3.3. Identification of DEGs and Functional Enrichment Analysis between the Control and Glutamine Supplementation Groups

**Figure 3:**
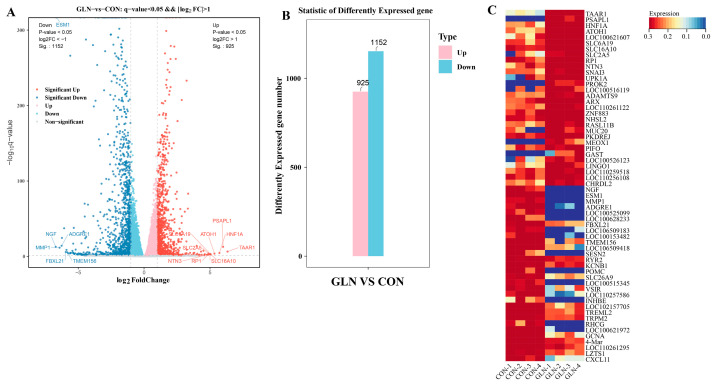
Identification of DEGs between the control (CON) and 2 mM glutamine (GLN) groups. (**A**) The volcano plot displays DEGs from GLN vs. CON. The x-axis represents the log2 (fold change) value, and the y-axis represents the log10 (*p*-value). Blue nodes represent down-regulated genes, while red nodes represent up-regulated genes. (**B**) Number of DEGs from GLN vs. CON. (**C**) Top 30 DEGs in the CON and GLN groups.

We evaluated the transcriptome changes using RNA-sequencing and detected DEGs between the groups to explore potential differences in transcriptomic expression for the control and glutamine supplementation groups. Compared with the control group, we identified that the glutamine supplementation group exhibits 925 up-regulated genes and 1152 down-regulated genes, as shown in the volcano plot and bar plot (Figure 3A,B). The top five up-regulated genes in the IPEC-J2 cells activated by glutamine are *TAAR1*, *PSAPL1*, *HNF1A*, *ATOH1*, *SLC16A10*, and *SLC6A19*. Conversely, the top five down-regulated genes consisted of *NGF*, *ESM1*, *MMP1*, *ADGRE1*, *FBXL21*, and *TMEM156* (Figure 3C).

**Figure 4:**
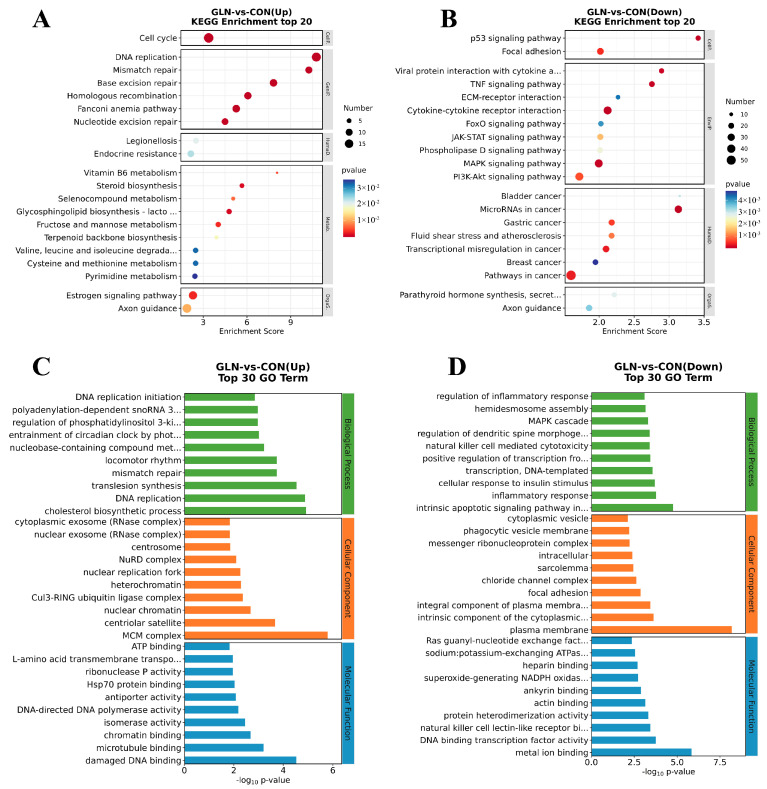
Identification of functional enrichment from DEGs. (**A**,**B**) KEGG functional enrichment for up-regulated DEGs and down-regulated DEGs. (**C**,**D**) GO pathways enriched for up-regulated DEGs and down-regulated DEGs.

For further investigation of notable signaling pathways through which glutamine promotes intestinal epithelial cell proliferation, DEGs between the control and glutamine supplementation groups were annotated using the KEGG and GO analyses. Five aspects of organismal systems were analyzed: metabolism, human diseases, genetic information processing, and cellular processes. The up-regulated DEGs show significant enrichment in the cell cycle, valine/leucine and isoleucine degradation, and cysteine and methionine metabolism. On the other hand, the down-regulated DEGs exhibit substantial enrichment in the p53 signaling pathway, focal adhesion, TNF signaling pathway, ECM-receptor interaction, foxO signaling pathway, JAK-STAT signaling pathway, phospholipase D signaling pathway, MAPK signaling pathway, and PI3K-AKT signaling pathway (Figure 4A,B).

We primarily explore biological processes, cellular components, and molecular functions during the GO analysis. The up-regulated enrichment includes DNA replication initiation, regulation of phosphatidylinositol 3-kinase activity, DNA replication, the minichromosome maintenance protein (*MCM*) complex, ATP binding, L-amino acid transmembrane transporter activity, and Hsp70 protein binding. Conversely, the down-regulated enrichment comprises regulation of the inflammatory response, the intrinsic apoptotic signaling pathway in response to endoplasmic reticulum stress, focal adhesion, and ras guanyl-nucleotide exchange factor activity (Figure 4C,D).

### 3.4. Unveiling the Vital Pathway and Genes Involved in Cell Proliferation and Anti-Inflammation

**Figure 5:**
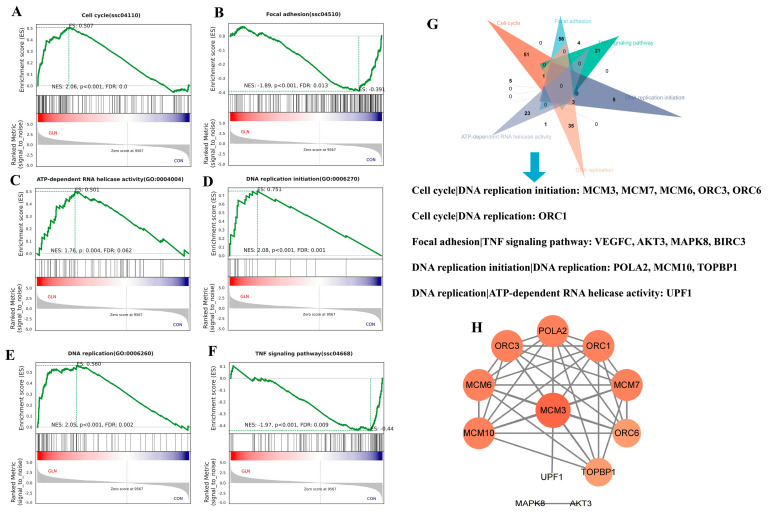
Identifying the crucial pathways and genes involved in cell proliferation and anti-inflammation. (**A**–**F**) Performing gene set enrichment analysis (GSEA) on IPEC-J2 transcriptomics data ranked from the glutamine treatment group (red) to the control group (blue) reveals significant enrichment in various pathways, including cell cycle, focal adhesion, TNF signaling pathway, DNA replication initiation, DNA replication, and ATP-dependent RNA helicase activity. (**G**) Venn plot illustrates the intersecting genes among the six crucial pathways. (**H**) The protein–protein association network of the intersection genes displays nodes representing the genes, with the size of each node indicating the number of connections (degree). Larger node sizes indicate a higher number of connections.

GSEA analysis was further performed to identify whether enriched gene sets showed a statistically significant difference between the groups. In the glutamine supplementation groups, there was a significant up-regulation of the cell cycle (normalized enrichment score (NES) = 2.06, *p* < 0.001), DNA replication initiation (NES = 2.08, *p* < 0.001), DNA replication (NES = 2.05, *p* < 0.001), and ATP-dependent RNA helicase activity (NES = 1.76, *p* < 0.01). Conversely, focal adhesion was down-regulated (NES = −1.89, *p* < 0.001) (Figure 5A–E). Notably, the glutamine supplementation group exhibited anti-inflammatory activity by downregulating the TNF signaling pathway (NES = 1.97, *p* < 0.001) (Figure 5F). These data provide some evidence to support the idea that glutamine promotes cell proliferation and inhibits inflammation through vital pathways, but this must be confirmed at the proteomic level and biochemically. Moreover, we identified intersecting genes (*MCM3*, *MCM7*, *MCM6*, *MCM10*, *ORC1*, *ORC3*, *ORC6*, *VEGFC*, *AKT3*, *MAPK8*, *BIRC3*, *POLA2*, *TOPBP1*, and *UPF1*) among the six crucial pathways (Figure 5G). The protein–protein association network of the intersecting genes highlighted the significance of DNA replication licensing factors (*MCM3*, *MCM6*, and *MCM10*) in promoting intestinal epithelial growth responding to glutamine (Figure 5H).

## 4. Discussion

The intestinal epithelium is one of the body’s fastest self-renewing tissues. Any disruptions to this process can trigger many diseases within and beyond the intestine, significantly impacting pig production efficiency [16,17]. Dietary glutamine undergoes rapid conversion into α-ketoglutarate, which is a crucial process supporting the accelerated renewal rate of the intestinal epithelium [18]. Previous research has implicated various factors in the regulation of glutamine-modulated growth of the intestinal epithelium, such as the amino acid transporter SLC7A5 [19], WNT signaling [9], Yes-associated protein [20], and the gut microbiome [21]. Indeed, some of the signals reported earlier are closely tied to cell cycle regulation and biofuel utilization. Consequently, unveiling the gene expression profile and identifying hub genes is imperative to comprehensively grasp the landscape and vital regulatory targets involved in glutamine-regulated intestinal epithelial homeostasis.

In this study, RNA sequencing was performed to investigate the transcriptome in IPEC-J2 cells of the control and glutamine supplementation groups, followed by principal component analysis, functional enrichment analysis, gene ontology analysis, KEGG analysis, gene set enrichment analysis, and protein–protein interaction analysis. Our results indicated that glutamine leads to diverse transcriptome changes in porcine intestinal epithelial cells, with 925 up-regulated genes and 1152 down-regulated genes. The up-regulated DEGs enrich KEGG pathways of the cell cycle, valine/leucine and isoleucine degradation, and cysteine and methionine metabolism. In contrast, the down-regulated DEGs enrich in KEGG pathways of the p53 signaling pathway, focal adhesion, TNF signaling pathway, ECM-receptor interaction, foxO signaling pathway, and JAK-STAT signaling pathway. In addition, the up-regulated DEGs enrich in GO terms of DNA replication initiation, regulation of phosphatidylinositol 3-kinase activity, DNA replication, MCM complex, ATP binding, L-amino acid transmembrane transporter activity, and Hsp70 protein binding. In contrast, the down-regulated DEGs enrich in GO terms of inflammatory response, intrinsic apoptotic signaling pathway in response to endoplasmic reticulum stress, focal adhesion, and ras guanyl-nucleotide exchange factor activity. Taken together, glutamine regulates signals about cell cycling and decreases inflammatory signals to support intestinal cell proliferation and stem cell expansion.

However, KEGG and GO analyses yielded paradoxical results. For instance, cell proliferation-facilitating signals from the MAPK and PI3K-AKT signaling pathways were enriched in down-regulated DEGs. Furthermore, the GO term “regulation of phosphatidylinositol 3-kinase activity” was enriched in up-regulated DEGs, whereas the KEGG pathway analysis showed enrichment of the PI3K-AKT signaling pathway in down-regulated DEGs. Relying solely on traditional enrichment analysis based on DEGs may be inadequate to fully explain the comprehensive gene regulation mechanisms [22]. Fortunately, GSEA analysis considers all genes in an experiment and maintains gene-gene connections, which can effectively identify dysregulated pathways [23]. Therefore, GSEA analysis was further performed to unveil the vital pathways and genes. Our results showed that pathways of the cell cycle, DNA replication initiation, DNA replication, and ATP-dependent RNA helicase activity are up-regulated. In contrast, focal adhesion and TNF signaling pathways are down-regulated, consistent with the major results of KEGG and GO analysis. The PPI analysis further highlights the significance of *MCM3*, *MCM6*, and *MCM10* in promoting intestinal epithelial growth in response to glutamine.

*MCMs* are the best-known protein family for maintaining normal cellular processes, especially DNA initiation and elongation [24]. At present, nine members of the *MCM* family, *MCM2–10*, have been identified in humans [25]. Recently, the *MCM* complex has been shown to act as a physical barrier, frequently constraining cohesion translocation, which restricts loop extrusion during the G1 phase and plays a crucial role in shaping the three-dimensional genome [26]. A replicative DNA helicase called *MCM2–7* hetero-hexamer forms mainly because of *MCM3*. MCM-binding proteins (MCMBPs) interact with the de novo synthesized *MCM3* to maintain the *MCM2–7* hexamer at a constantly high level [27]. Wang et al. reported that Hippo-Yes-associated protein (YAP) binds to the promoter of *MCM6*, inducing its transcription and activating PI3K/Akt/GSK3β signaling to support cell growth. Conversely, the knockdown of *MCM6* suppresses cell proliferation, migration, and organoid expansion [28]. In addition, *MCM6* recruits ubiquitin ligase E3A (*UBE3A*) to enhance the ubiquitination and degradation of the p53 protein, which can facilitate the aggressiveness of cells [29]. *MCM10* dysfunction leads to telomerase shortening by accumulating aberrant replication forks enriched with single-stranded DNA [30]. A recent study demonstrated that *MCM10* maintains the integrity of the genome in the proliferating hemogenic endothelium and then facilitates the specification of hematopoietic stem cells from the hemogenic endothelium. Moreover, *MCM10* deficiency in embryos contributes to the apoptosis of hematopoietic stem cells, which can be rescued by knocking down p53 [31].

Improvements are also needed in the following studies: First, enough intestinal organoids in series time points, rather than IPEC-J2 cells, should be applied to explore critical targets and cell–cell communications responding to glutamine exposure using recent advanced techniques such as single-cell transcriptomics and spatial transcriptomics. Moreover, the characteristics of the obtained targets or cell populations in intestinal mucosal functions and gut health must be approved in vitro and in vivo.

## 5. Conclusions

In conclusion, glutamine regulates gene expression profiles in porcine intestinal epithelial cells. Among many DEGs, glutamine may upregulate MCM3 and MCM6 to progress the MCM2–7 hexamer, followed by increased PI3K/Akt signaling, resulting in the proliferation of porcine intestinal cells. Additionally, a high level of MCM10 evaluated by glutamine ensures the stability of the genome and the activity of telomerase to facilitate stem cell expansion. Along with promoting intestinal epithelial proliferation and stem cell expansion, glutamine may also reduce inflammation brought on by TNF signaling, JAK-STAT signaling, and p53-induced apoptosis. Further studies are required to validate the findings, which would disclose crucial targets for regulating intestinal mucosal function and gut health.

## Data Availability

The data supporting the findings of the present study are available from the corresponding author upon reasonable request.

## References

[1] Pentinmikko N., Iqbal S., Mana M., Andersson S., Cognetta A.B., Suciu R.M., Roper J., Luopajärvi K., Markelin E., Gopalakrishnan S. (2019). Notum produced by Paneth cells attenuates regeneration of aged intestinal epithelium. Nature.

[2] Xian C., Zhang J., Zhao S., Li X.G. (2023). Gut-on-a-chip for disease models. J. Tissue Eng..

[3] Gehart H., Clevers H. (2019). Tales from the crypt: New insights into intestinal stem cells. Nat. Rev. Gastroenterol. Hepatol..

[4] Zhu P., Lu T., Wu J., Fan D., Liu B., Zhu X., Guo H., Du Y., Liu F., Tian Y. (2022). Gut microbiota drives macrophage-dependent self-renewal of intestinal stem cells via niche enteric serotonergic neurons. Cell Res..

[5] Newsholme P., Procopio J., Lima M.M., Pithon-Curi T.C., Curi R. (2003). Glutamine and glutamate--their central role in cell metabolism and function. Cell Biochem. Funct..

[6] Yoo H.C., Yu Y.C., Sung Y., Han J.M. (2020). Glutamine reliance in cell metabolism. Exp. Mol. Med..

[7] Xiao D., Zeng L., Yao K., Kong X., Wu G., Yin Y. (2016). The glutamine-alpha-ketoglutarate (AKG) metabolism and its nutritional implications. Amino Acids.

[8] Sakai R., Ooba Y., Watanabe A., Nakamura H., Kawamata Y., Shimada T., Takumi A., van Goudoever J.B., Narita T. (2020). Glutamate metabolism in a human intestinal epithelial cell layer model. Amino Acids.

[9] Tian J., Li Y., Bao X., Yang F., Tang X., Jiang Q., Yang C., Yin Y., Yao K. (2023). Glutamine boosts intestinal stem cell-mediated small intestinal epithelial development during early weaning: Involvement of WNT signaling. Stem Cell Rep..

[10] Tran T.Q., Hanse E.A., Habowski A.N., Li H., Ishak Gabra M.B., Yang Y., Lowman X.H., Ooi A.M., Liao S.Y., Edwards R.A. (2020). alpha-Ketoglutarate attenuates Wnt signaling and drives differentiation in colorectal cancer. Nat. Cancer.

[11] Qin Y.C., Zhou J.Y., Zhu M., Zan G.X., Gao C.Q., Yan H.C., Li X.G., Wang X.Q. (2022). L-glutamate requires β-catenin signalling through Frizzled7 to stimulate porcine intestinal stem cell expansion. Cell Mol. Life Sci..

[12] Zhu M., Qin Y.C., Gao C.Q., Yan H.C., Li X.G., Wang X.Q. (2019). Extracellular Glutamate-Induced mTORC1 Activation via the IR/IRS/PI3K/Akt Pathway Enhances the Expansion of Porcine Intestinal Stem Cells. J. Agric. Food Chem..

[13] Li X.G., Sui W.G., Gao C.Q., Yan H.C., Yin Y.L., Li H.C., Wang X.Q. (2016). L-Glutamate deficiency can trigger proliferation inhibition via down regulation of the mTOR/S6K1 pathway in pig intestinal epithelial cells. J. Anim. Sci..

[14] Ye J.L., Gao C.Q., Li X.G., Jin C.L., Wang D., Shu G., Wang W.C., Kong X.F., Yao K., Yan H.C. (2016). EAAT3 promotes amino acid transport and proliferation of porcine intestinal epithelial cells. Oncotarget.

[15] Li X.G., Zhu M., Chen M.X., Fan H.B., Fu H.L., Zhou J.Y., Zhai Z.Y., Gao C.Q., Yan H.C., Wang X.Q. (2019). Acute exposure to deoxynivalenol inhibits porcine enteroid activity via suppression of the Wnt/β-catenin pathway. Toxicol. Lett..

[16] Li X.G., Chen M.X., Zhao S.Q., Wang X.Q. (2022). Intestinal Models for Personalized Medicine: From Conventional Models to Microfluidic Primary Intestine-on-a-chip. Stem Cell Rev. Rep..

[17] Zhou J.Y., Huang D.G., Zhu M., Gao C.Q., Yan H.C., Li X.G., Wang X.Q. (2020). Wnt/β-catenin-mediated heat exposure inhibits intestinal epithelial cell proliferation and stem cell expansion through endoplasmic reticulum stress. J. Cell Physiol..

[18] Wong C.C., Xu J., Bian X., Wu J.L., Kang W., Qian Y., Li W., Chen H., Gou H., Liu D. (2020). In Colorectal Cancer Cells with Mutant KRAS, SLC25A22-Mediated Glutaminolysis Reduces DNA Demethylation to Increase WNT Signaling, Stemness, and Drug Resistance. Gastroenterology.

[19] Najumudeen A.K., Ceteci F., Fey S.K., Hamm G., Steven R.T., Hall H., Nikula C.J., Dexter A., Murta T., Race A.M. (2021). The amino acid transporter SLC7A5 is required for efficient growth of KRAS-mutant colorectal cancer. Nat. Genet..

[20] Chen X., Zhang P.Y., Zhang Y.J., Fan S.J., Wei Y., Yang Z.F., Wang F.C., Peng X. (2023). Potential Effect of Glutamine in the Improvement of Intestinal Stem Cell Proliferation and the Alleviation of Burn-Induced Intestinal Injury via Activating YAP: A Preliminary Study. Nutrients.

[21] Holthausen J.S., Schregel J., Sciascia Q.L., Li Z.Y., Tuchscherer A., Vahjen W., Metges C.C., Zentek J. (2022). Effects of Oral Glutamine Supplementation, Birthweight and Age on Colonic Morphology and Microbiome Development in Male Suckling Piglets. Microorganisms.

[22] Yu X.T., Zeng T., Li G.J. (2015). Integrative enrichment analysis: A new computational method to detect dysregulated pathways in heterogeneous samples. BMC Genom..

[23] Subramanian A., Tamayo P., Mootha V.K., Mukherjee S., Ebert B.L., Gillette M.A., Paulovich A., Pomeroy S.L., Golub T.R., Lander E.S. (2005). Gene set enrichment analysis: A knowledge-based approach for interpreting genome-wide expression profiles. Proc. Natl. Acad. Sci. USA.

[24] Wang Y.F., Chen H.R., Zhang J.L., Cheng A.S.L., Yu J., To K.F., Kang W. (2020). MCM family in gastrointestinal cancer and other malignancies: From functional characterization to clinical implication. Biochim. Biophys. Acta Rev. Cancer.

[25] Lei Y., Wang S., Liu J., Yan W., Han P., Tian D. (2021). Identification of MCM family as potential therapeutic and prognostic targets for hepatocellular carcinoma based on bioinformatics and experiments. Life Sci..

[26] Dequeker B.J.H., Scherr M.J., Brandão H.B., Gassler J., Powell S., Gaspar I., Flyamer I.M., Lalic A., Tang W., Stocsits R. (2022). MCM complexes are barriers that restrict cohesin-mediated loop extrusion. Nature.

[27] Saito Y., Santosa V., Ishiguro K., Kanemaki M.T. (2022). MCMBP promotes the assembly of the MCM2-7 hetero-hexamer to ensure robust DNA replication in human cells. eLife.

[28] Wang Y., Chen H., Liu W., Yan H., Zhang Y., Cheung A.H.K., Zhang J., Chen B., Liang L., Zhou Z. (2022). MCM6 is a critical transcriptional target of YAP to promote gastric tumorigenesis and serves as a therapeutic target. Theranostics.

[29] Zhang X., Bian S., Ni Y., Zhou L., Yang C., Zhang C., Sun X., Xu N., Xu S., Wang Y. (2023). Minichromosome maintenance protein family member 6 mediates hepatocellular carcinoma progression by recruiting UBE3A to induce P53 ubiquitination. Int. J. Biol. Macromol..

[30] Baxley R.M., Leung W., Schmit M.M., Matson J.P., Yin L., Oram M.K., Wang L., Taylor J., Hedberg J., Rogers C.B. (2021). Bi-allelic MCM10 variants associated with immune dysfunction and cardiomyopathy cause telomere shortening. Nat. Commun..

[31] Cacialli P., Dogan S., Linnerz T., Pasche C., Bertrand J.Y. (2023). Minichromosome maintenance protein 10 (mcm10) regulates hematopoietic stem cell emergence in the zebrafish embryo. Stem Cell Rep..

